# Serum alkaline phosphatase levels at admission are associated with unfavorable prognosis in acute ischemic stroke patients undergoing endovascular thrombectomy

**DOI:** 10.3389/fneur.2026.1738653

**Published:** 2026-02-17

**Authors:** Xiaohong Tang, Qingyun Li, Wei Zhang

**Affiliations:** 1 Department of Neurology, Hongze District People’s Hospital, Huaian, Jiangsu, China; 2 Department of Neurology, The Affiliated Hospital of Xuzhou Medical University, Xuzhou, Jiangsu, China

**Keywords:** acute ischemic stroke, alkaline phosphatase, endovascular thrombectomy, linear association, prognosis

## Abstract

**Background:**

Limited data exist on the association between alkaline phosphatase (ALP) levels and outcomes in acute ischemic stroke patients undergoing endovascular thrombectomy. This study aimed to evaluate the relationship between serum ALP levels at admission and unfavorable prognosis following endovascular thrombectomy.

**Methods:**

This retrospective study included patients who underwent mechanical thrombectomy for acute ischemic stroke (AIS) within 24 h of symptom onset at the Affiliated Hospital of Xuzhou Medical University between October 2018 and May 2025. Blood samples were collected upon admission in the emergency room. Unfavorable prognosis was defined as a modified Rankin Scale score of 3–6 at 90 days. Logistic regression analyses were conducted to examine the relationship between ALP levels and unfavorable prognosis.

**Results:**

Of the 385 enrolled patients, 209 (54.3%) experienced an unfavorable prognosis. These patients exhibited significantly higher serum ALP levels (83.8 ± 29.5 U/L versus 76.1 ± 27.9 U/L; *p* = 0.009) compared to those with a favorable prognosis. A significant positive association was found between ALP levels (per 10-unit increase) and unfavorable prognosis (OR: 1.17, 95% CI: 1.06–1.29; *p* = 0.002) after adjusting for multiple variables. Patients in the highest ALP tertile had significantly higher odds of an unfavorable prognosis compared to those in the lowest tertile (OR: 3.17, 95% CI: 1.61–6.24; *p* = 0.001). The restricted cubic spline indicated a positive linear relationship between ALP levels and unfavorable prognosis (*p* for non-linearity = 0.461). The association between ALP levels and unfavorable prognosis remained stable across different subgroups (all *p* for interaction > 0.05).

**Conclusion:**

Our findings demonstrate a positive association between serum ALP levels at admission and unfavorable prognosis in patients with AIS who underwent endovascular thrombectomy.

## 1 Introduction

Acute ischemic stroke (AIS) with large vessel occlusion (LVO) is a leading cause of death and disability worldwide (1). The primary objective is to restore cerebral blood flow using reperfusion therapies, such as intravenous alteplase and endovascular thrombectomy (ET) (2). Despite successful recanalization in more than 80% of AIS patients undergoing ET, functional dependence and mortality remain high, affecting about half of the cases (3). Several clinical and angiographic factors, including advanced age, hyperglycemia, higher baseline NIHSS scores, larger infarct volumes, longer onset-to-recanalization times, and poor collateral status, are linked to unfavorable outcomes (4).

Alkaline phosphatase (ALP) is a ubiquitous enzyme present in multiple isoenzymes, primarily found in the liver, bone, intestine, and kidney (5). Although its exact physiological function is not fully understood, ALP is believed to play roles in bone calcification, intestinal phosphate transport, and membrane transport processes (6). Serum ALP levels are commonly used as indicators of liver and renal damage (7, 8). Moreover, elevated serum ALP levels are associated with an increased risk of cardiovascular disease (CVD) (9), peripheral arterial disease (PAD) (10), and ischemic stroke (11). While the relationship between ALP levels and stroke prognosis is inconsistent (12), limited data focuses on patients undergoing thrombectomy. This study aims to investigate whether ALP levels at admission are associated with prognosis in AIS patients treated with thrombectomy.

## 2 Materials and methods

### 2.1 Study design and population

This study retrospectively reviewed cases of patients with AIS who underwent ET at our institution from October 2018 to May 2025. Subjects were excluded based on the following criteria: (1) occlusion in the posterior circulation or anterior cerebral artery (ACA); (2) not treated with thrombectomy due to tortuous vessels, non-large vessel occlusion, revascularization after thrombolysis, chronic occlusion, isolated intra-arterial thrombolysis, or isolated angioplasty; (3) pre-modified Rankin Scale (mRS) > 2; (4) incomplete laboratory tests or follow-up data; or (5) unavailability of non-contrast CT or digital subtraction angiography (DSA) images. The baseline characteristics of included and excluded participants are presented in Supplementary Table S1. This study was approved by the Ethics Committee of the Affiliated Hospital of Xuzhou Medical University, and written informed consent was waived due to its retrospective nature.

### 2.2 Data collection

Blood samples, including a complete blood count and standard biochemistry profile, were collected upon admission to the emergency room. Clinical and radiological data were gathered, including sex, age, medical history [hypertension (EH), diabetes mellitus (DM), dyslipidemia, atrial fibrillation (AF), coronary artery disease (CAD)], stroke subtype based on the Trial of Org 10172 in Acute Stroke Treatment (TOAST) classification (13), baseline National Institutes of Health Stroke Scale (NIHSS) score, baseline Alberta Stroke Program Early CT Score (ASPECTS), treatment with intravenous thrombolysis, occlusion site, tandem occlusion, collateral status, and onset to puncture time (OPT). Additionally, procedural characteristics such as procedure duration, anesthesia type, distal embolism, residual stenosis, first-line treatment modality, number of maneuvers, rescue therapy, recanalization status, and intracranial hemorrhage were recorded. Three-month functional outcomes were assessed by the mRS via trained telephone interviews, with scores of 3–6 indicating unfavorable prognosis (14). All CT and DSA images were independently evaluated by two experienced interventional neuroradiologists blinded to clinical information, with an inter-rater reliability *κ* value of 0.77.

### 2.3 Statistical analysis

Patients were divided into two groups, unfavorable prognosis and favorable prognosis. Continuous variables with normal distribution were expressed as mean ± standard deviation (SD), while skewed variables were presented as medians with interquartile ranges (IQRs). Categorical variables were expressed as frequencies and percentages (%). Differences between the two groups were assessed using the Student’s *t*-test, Mann–Whitney U-test, or chi-square test as appropriate. Logistic regression analyses were performed to explore the association between ALP levels and unfavorable prognosis, with odds ratios (ORs) and 95% confidence intervals (CIs) calculated for three sequentially adjusted models. Confounding was assessed by integrating prior scientific knowledge and descriptive statistical insights from our study cohort, with the aid of directed acyclic graphs (DAGs) (Supplementary Figure S1). Model 1 adjusted for sex, age, EH, DM, baseline ASPECTS, eGFR, ALT, AST, and WBC; Model 2 further adjusted for AF, baseline NIHSS, occlusion site, collateral status, OPT, first-line treatment, and IV thrombolysis; Model 3 further adjusted for procedure time, mTICI, maneuvers, and any ICH. To improve the robustness of the results, the ALP was transformed as a categorical variable in the logistic regression models and a trend test was performed.

A restricted cubic spline (RCS) was used to assess the dose–response relationship between ALP and unfavorable prognosis. The curve was adjusted based on covariables in Model 3, using three knots (10th, 50th, and 90th percentiles) of ALP distribution. Subgroup analyses were conducted to investigate the association between ALP and unfavorable prognosis across different populations, including sex, age, stroke causes, baseline NIHSS, baseline ASPECTS, IV thrombolysis, and occlusion site.

A *p*-value of less than 0.05 indicated significance in two-tailed tests. All analyses and visualizations were performed using R (version 4.3.1; The R Foundation, Vienna, Austria) and Free Statistics software (version 1.9.2; Beijing Free Clinical Medical Technology Co., Ltd., Beijing, China).

## 3 Results

### 3.1 Study population and clinical characteristics

**Figure 1:**
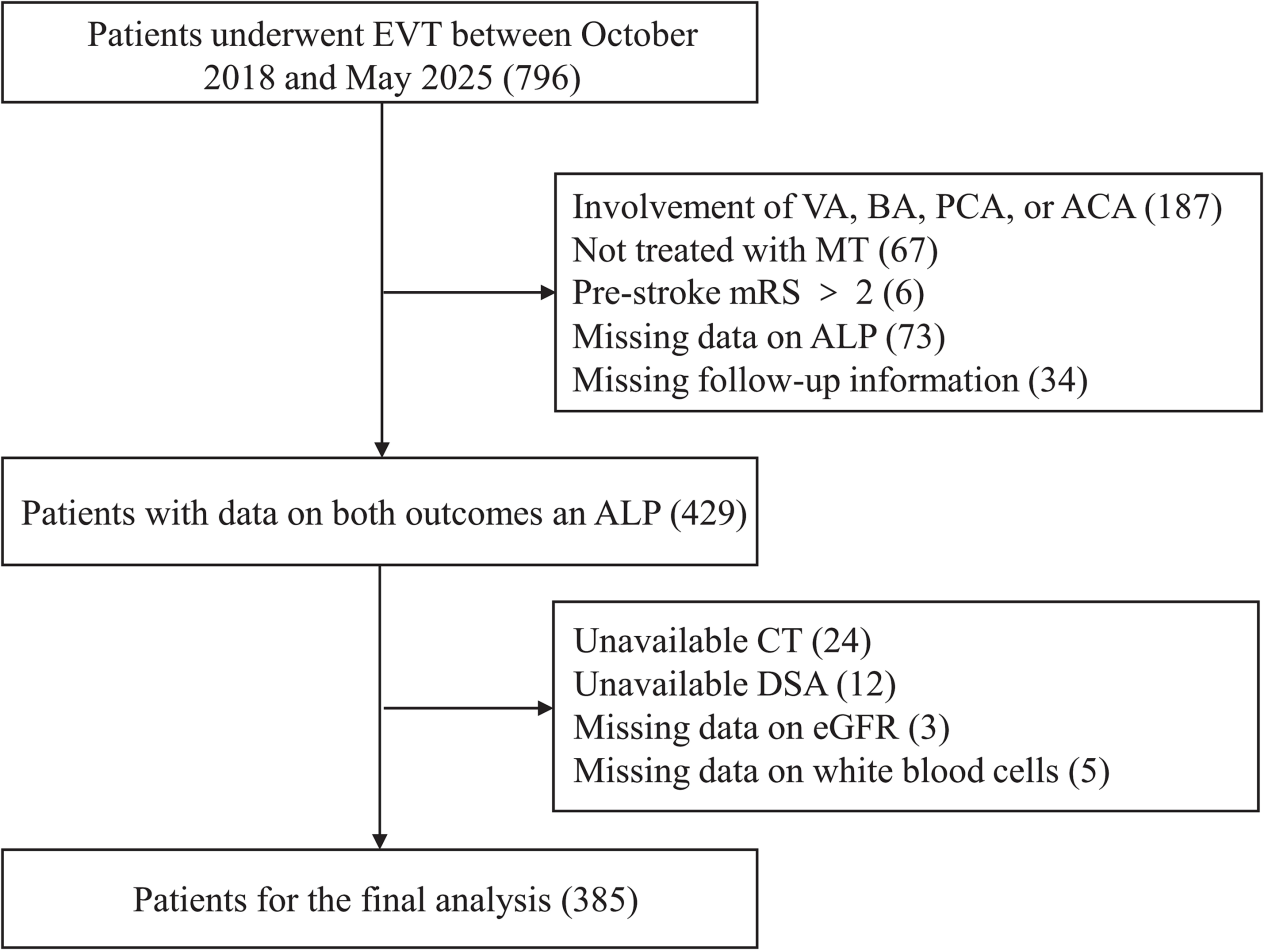
Flowchart of patient selection.

**Figure 2:**
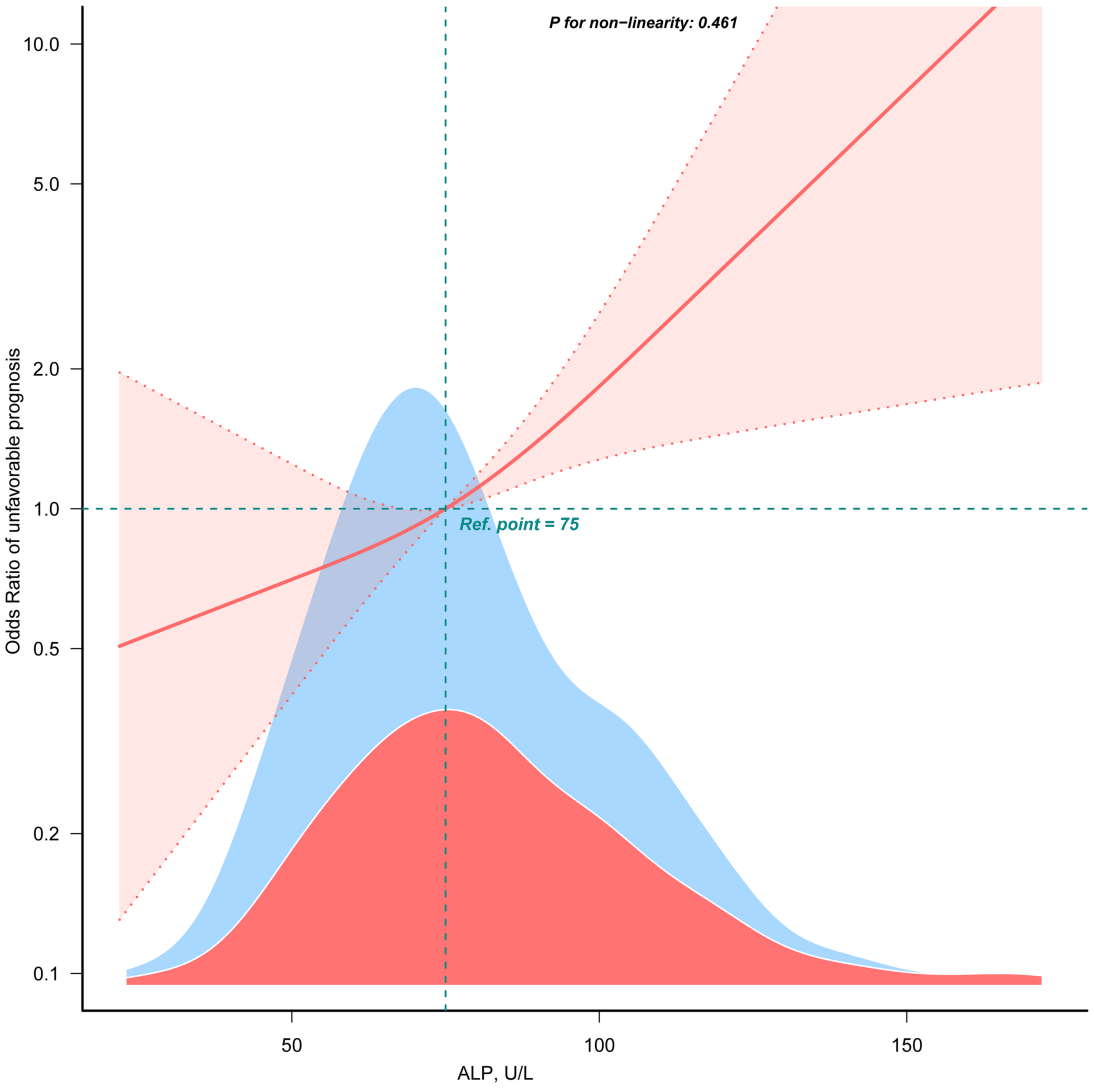
Proportion of patients with unfavorable prognosis stratified by the tertiles of ALP.

Of the 796 patients who underwent endovascular thrombectomy at our institution, 385 were included in this study (Figure 1). Table 1 presents the clinical, angiographic, and laboratory characteristics of patients, categorized by clinical outcomes. Among the 385 patients, 245 (63.6%) were men, with a mean age of 66.6 years (SD = 12.5). A total of 209 patients (54.3%) had an unfavorable prognosis. The mean ALP level was 80.3 U/L (SD = 29.0). Women were more likely to experience an unfavorable prognosis. Patients with an unfavorable prognosis were generally older and had a higher prevalence of atrial fibrillation (AF). They also presented with higher baseline NIHSS scores, lower baseline ASPECTS, and more frequent involvement of the ICA. Additionally, these patients had poorer collateral status, longer OPT, and longer procedure time. They experienced lower revascularization rates, more frequently used aspiration as the first-line treatment, required more maneuvers, and had a higher incidence of intracerebral hemorrhage (ICH). In terms of laboratory values, patients with an unfavorable prognosis were more likely to have higher ALP levels and lower eGFR levels. There was a trend of increasing unfavorable prognosis with rising ALP levels, with the proportions being 44.2, 54.9, and 62.9%, respectively (Figure 2).

**Table 1:** Comparison of baseline demographic, clinical, and procedure characteristics in patients with different prognosis.

Characteristics	Total (*n* = 385)	Unfavorable prognosis (*n* = 209)	Favorable prognosis (*n* = 176)	*p*-value
Sex, *n* (%)				0.033
Male	245 (63.6)	123 (58.9)	122 (69.3)	
Female	140 (36.4)	86 (41.1)	54 (30.7)	
Age, years, mean ± SD	66.6 ± 12.5	69.3 ± 11.5	63.4 ± 13.0	<0.001
EH, *n* (%)	196 (50.9)	110 (52.6)	86 (48.9)	0.461
DM, *n* (%)	78 (20.3)	47 (22.5)	31 (17.6)	0.236
Dyslipidemia, *n* (%)	24 (6.2)	11 (5.3)	13 (7.4)	0.391
AF, *n* (%)	178 (46.2)	108 (51.7)	70 (39.8)	0.020
CAD, *n* (%)	53 (13.8)	33 (15.8)	20 (11.4)	0.209
Cause, *n* (%)				0.224
Atherosclerotic	159 (41.3)	78 (37.3)	81 (46)	
Cardioembolic	173 (44.9)	100 (47.8)	73 (41.5)	
Others	53 (13.8)	31 (14.8)	22 (12.5)	
Baseline NIHSS, mean ± SD	17.9 ± 8.0	19.9 ± 8.1	15.5 ± 7.1	<0.001
Baseline ASPECTS, mean ± SD	7.7 ± 1.5	7.4 ± 1.6	8.1 ± 1.3	<0.001
IV thrombolysis, *n* (%)	133 (34.5)	71 (34)	62 (35.2)	0.796
Occlusion site, *n* (%)				<0.001
ICA	129 (33.5)	87 (41.6)	42 (23.9)	
M1	240 (62.3)	116 (55.5)	124 (70.5)	
M2	16 (4.2)	6 (2.9)	10 (5.7)	
Tandem occlusion, *n* (%)	26 (6.8)	14 (6.7)	12 (6.8)	0.963
Good collateral status, *n* (%)	237 (61.6)	108 (51.7)	129 (73.3)	<0.001
Unknown time, *n* (%)	85 (22.1)	50 (23.9)	35 (19.9)	0.341
OPT, minutes, mean ± SD	278.6 ± 125.3	291.5 ± 134.8	263.3 ± 111.6	0.028
OPT, *n* (%)				0.638
0–6 h	331 (86.0)	179 (85.6)	152 (86.4)	
6–12 h	52 (13.5)	28 (13.4)	24 (13.6)	
12–24 h	2 (0.5)	2 (1)	0 (0)	
Procedure time, minutes, mean ± SD	82.0 ± 48.2	92.6 ± 51.4	69.5 ± 40.8	<0.001
General anesthesia, *n* (%)	69 (17.9)	44 (21.1)	25 (14.2)	0.081
mTICI 2b-3, *n* (%)	317 (82.3)	155 (74.2)	162 (92)	<0.001
Distal embolism, *n* (%)	115 (29.9)	67 (32.1)	48 (27.3)	0.307
Residual stenosis, *n* (%)	56 (14.5)	26 (12.4)	30 (17)	0.202
Rescue therapy, *n* (%)	31 (8.1)	20 (9.6)	11 (6.2)	0.233
First-line treatment, *n* (%)				0.037
SR	330 (85.7)	172 (82.3)	158 (89.8)	
CA	55 (14.3)	37 (17.7)	18 (10.2)	
Maneuvers, passes, mean ± SD	1.6 ± 0.9	1.8 ± 1.0	1.5 ± 0.7	<0.001
Any ICH, *n* (%)	171 (44.4)	116 (55.5)	55 (31.2)	<0.001
ALP, U/L, mean ± SD	80.3 ± 29.0	83.8 ± 29.5	76.1 ± 27.9	0.009
AST, U/L, median (IQR)	26.0 (20.0, 33.0)	26.0 (20.0, 33.0)	26.0 (20.0, 32.0)	0.418
ALT, U/L, median (IQR)	23.0 (17.0, 36.0)	22.0 (16.0, 36.0)	24.0 (17.0, 35.0)	0.504
eGFR, mL/min/1.73 m^2^, mean ± SD	99.7 ± 15.9	97.3 ± 15.4	102.7 ± 16.0	<0.001
WBC, × 10^9^/L, mean ± SD	8.5 ± 2.9	8.6 ± 3.0	8.4 ± 2.8	0.552

EH, hypertension; DM, diabetes mellitus; AF, atrial fibrillation; CAD, coronary artery disease; NIHSS, National Institutes of Health Stroke Scale; ASPECTS, Alberta Stroke Program Early CT Score; IV, intravenous; ICA, internal carotid artery; M, middle cerebral artery; OPT, onset to puncture time; mTICI, modified Thrombolysis in Cerebral Infarction; SR, stent retriever; CA, contact aspiration; ICH, intracranial cerebral hemorrhage; ALP, alkaline phosphatase; ALT, alanine aminotransferase; AST, aspartate aminotransferase; eGFR, estimated glomerular filtration rate; WBC, white blood cell.

### 3.2 Associations between ALP and unfavorable prognosis

**Figure 3:**
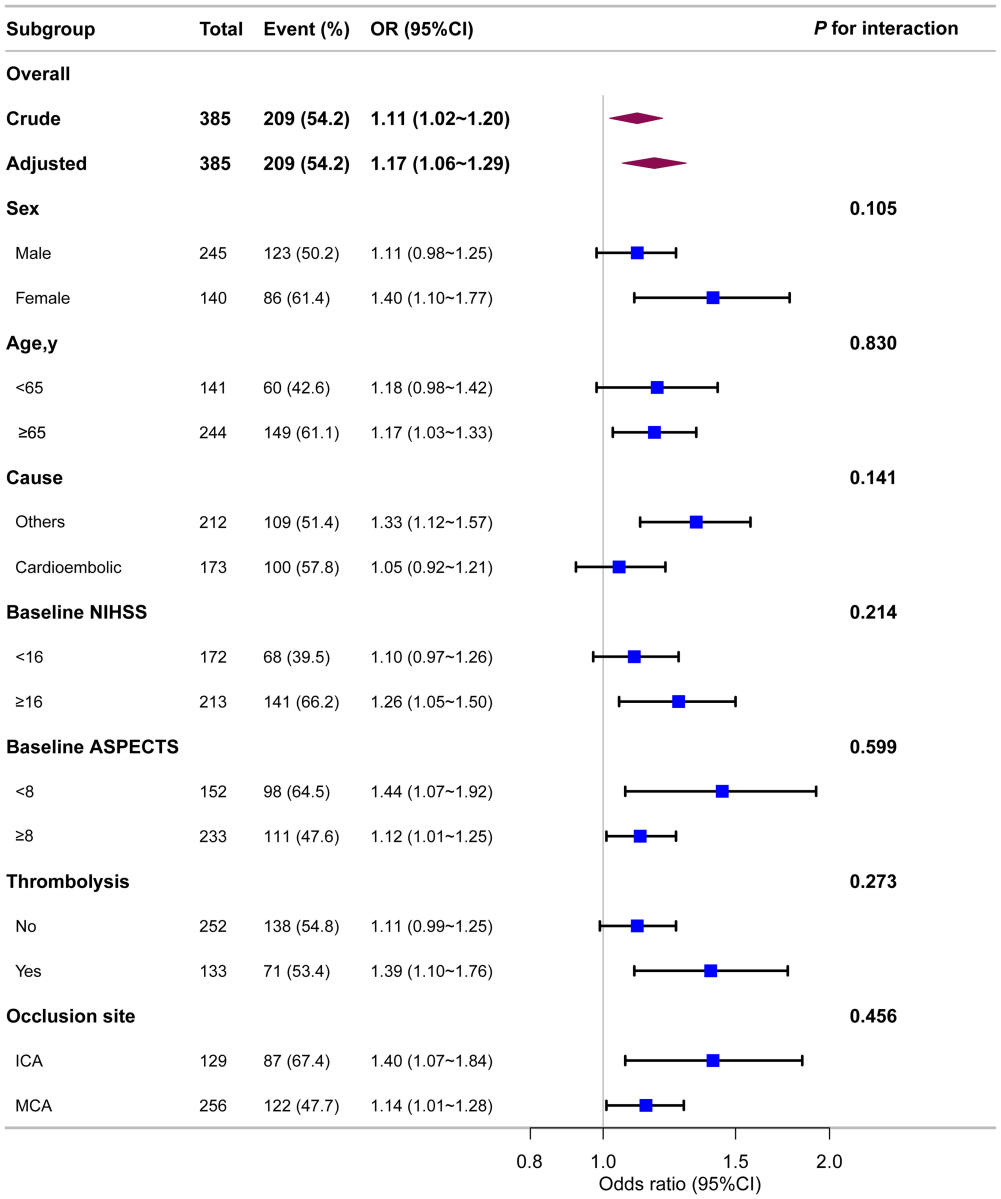
The linear association between ALP and unfavorable prognosis. Data were fit by a multivariable logistic regression model based on restricted cubic spline. Data were adjusted for sex, age, AF, baseline NIHSS, baseline ASPECTS, occlusion site, collateral status, OPT, procedure time, mTICI, first-line treatment, maneuvers, any ICH, eGFR, EH, DM, IV thrombolysis, ALT, AST, and WBC (Model 3). Here the median ALP was defined as the reference standard. Solid and dashed lines indicate the predicted value and 95% CI. Only 99% of the data is shown.

Table 2 showed the association between ALP levels and unfavorable outcomes following thrombectomy. When ALP was analyzed as a continuous variable, each 10-U/L increase was associated with an approximately 17% higher risk of an unfavorable outcome (Model 3, OR: 1.17, 95% CI: 1.06–1.29; *p* = 0.002). When ALP was categorized into tertiles, patients in the highest tertile (T3) had significantly higher odds of an unfavorable prognosis than those in the lowest tertile (T1) (OR: 3.17, 95% CI: 1.61–6.24; *p* = 0.001), while the middle tertile (T2) showed an elevated but not statistically significant odds (OR: 1.59, 95% CI: 0.84–3.02; *p* = 0.153). A linear relationship was observed between ALP and unfavorable prognosis (non-linearity: *p* = 0.461) using the restricted cubic spline model (Figure 3).

**Table 2:** Multivariable analyses for the association between ALP levels and unfavorable prognosis.

ALP	Model 1			Model 2		Model 3	
	No.	OR (95%CI)	*p-*value	OR (95%CI)	*p-*value	OR (95%CI)	*p-*value
Per 10-unit increase	385	1.10 (1.01 ~ 1.20)	0.033	1.13 (1.03 ~ 1.24)	0.010	1.17 (1.06 ~ 1.29)	0.002
Subgroup (tertiles)							
T1	120	1 (Ref)		1 (Ref)		1 (Ref)	
T2	133	1.51 (0.87 ~ 2.61)	0.145	1.53 (0.84 ~ 2.78)	0.160	1.59 (0.84 ~ 3.02)	0.153
T3	132	2.20 (1.25 ~ 3.89)	0.007	2.80 (1.49 ~ 5.25)	0.001	3.17 (1.61 ~ 6.24)	0.001
*p* for trend			0.007		0.001		0.001

Model 1 adjusted for sex, age, EH, DM, baseline ASPECTS, eGFR, ALT, AST, and WBC; Model 2 further adjusted for AF, baseline NIHSS, occlusion site, collateral status, OPT, first-line treatment, and IV thrombolysis; Model 3 further adjusted for procedure time, mTICI, maneuvers, and any ICH. ALP, alkaline phosphatase; OR, odds ratio; CI, confidence interval; T, tertiles.

### 3.3 Subgroup analyses

**Figure 4:**
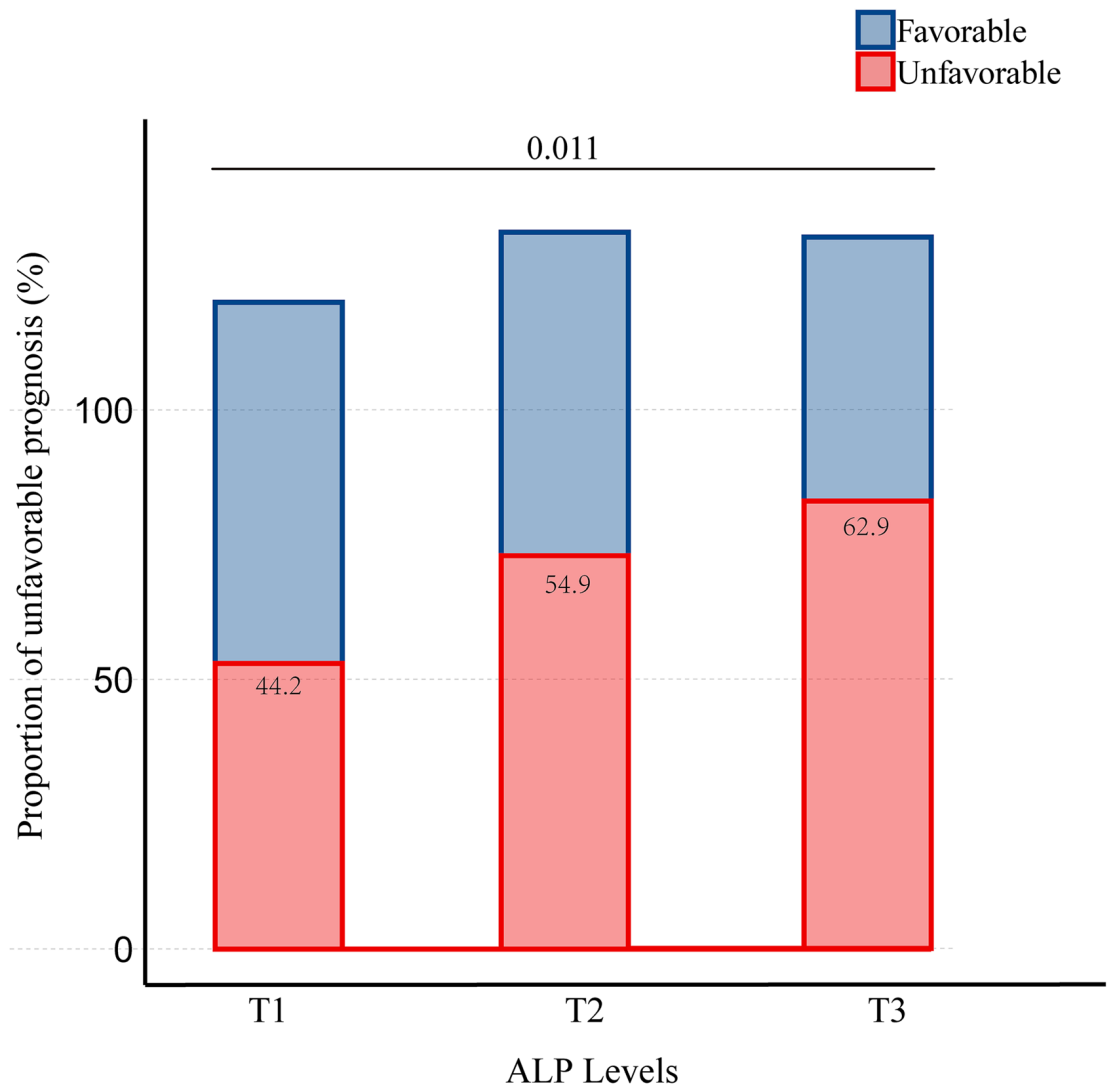
Association between ALP and unfavorable prognosis in different subgroups. Each stratification was adjusted for all variables (sex, age, AF, baseline NIHSS, baseline ASPECTS, occlusion site, collateral status, OPT, procedure time, mTICI, first-line treatment, maneuvers, any ICH, eGFR, EH, DM, IV thrombolysis, ALT, AST, and WBC) except the stratification factor itself.

As shown in Figure 4, stratified analyses across multiple subgroups revealed no significant interactions in any subgroup when stratified by sex, age, stroke causes, baseline NIHSS, baseline ASPECTS, IV thrombolysis, or occlusion site (all *p* > 0.05).

## 4 Discussion

We observed a positive correlation between elevated ALP levels and an increased risk of unfavorable prognosis in AIS patients with anterior circulation large-vessel occlusion undergoing ET. Specifically, the odds of an unfavorable prognosis increased by 17% for each 10-unit rise in ALP levels, with the association remaining robust across subgroup analyses.

ALP is crucial for the formation of calcifying nanoparticles *in vitro*, potentially contributing to pathological calcification (15). Recent studies have identified a positive association between ALP and early arteriosclerosis (16). In coronary artery disease patients, elevated serum ALP levels have been linked to increased mortality, myocardial infarction, and stent thrombosis (17). Furthermore, elevated ALP levels correlate with peripheral arterial disease (PAD), independent of traditional risk factors (10).

In the brain, ALP is expressed on cerebral endothelial cells, showing a developmental pattern with minimal expression before 28 weeks of gestation and adult-like patterns thereafter (18, 19). It is involved in insulin transport across the blood–brain barrier (BBB) and may regulate agmatine levels in the brain (20, 21). Previous research has investigated the association between ALP levels and stroke, showing that higher ALP levels correlate with a greater risk of cerebral small vessel disease, including white matter hyperintensities and silent lacunar infarcts (22, 23). Some studies found no significant correlation between ALP and NIHSS scores or functional outcomes (11), while others observed a J-shaped relationship between ALP levels and 3-month mortality in AIS patients (24). Consistently, a Korean study found increased serum ALP levels were an independent predictor of all-cause and vascular death after ischemic or hemorrhagic stroke (12). In terms of in-hospital mortality, a significant linear association between ALP and death was observed (25). In patients undergoing intravenous thrombolysis, elevated ALP levels were associated with poor outcomes (26). A recent study found that elevated serum ALP levels at admission were independently associated with futile recanalization in AIS patients treated with ET (27).

The underlying mechanisms of the association between ALP levels and unfavorable prognosis are not fully understood, but several hypotheses exist. First, elevated ALP levels may be linked to accelerated vascular calcification. Transgenic overexpression of tissue-nonspecific ALP in vascular endothelium has resulted in generalized arterial calcification and cardiovascular dysfunction in mice (28). Inflammatory cells, particularly macrophages, contribute to vascular calcification by producing factors that induce ALP expression in vascular smooth muscle cells (29, 30). Additionally, inhibition of tissue-nonspecific ALP has been proposed as a potential therapeutic strategy to prevent vascular calcification (28). Second, ALP may be associated with systemic inflammation. Serum ALP activity is significantly elevated in acute sepsis and other inflammatory conditions (31). ALP levels have been positively correlated with inflammatory markers like C-reactive protein and leukocyte counts (32). It was assumed that the increased expression of ALP could be a cellular response to inflammatory stimuli (33). ALP’s anti-inflammatory properties, attributed to its ability to dephosphorylate and detoxify lipopolysaccharide and convert extracellular ATP to adenosine (34, 35), might result in an unfavorable host defense response (36). Third, ALP plays a crucial role in maintaining blood–brain barrier (BBB) function and modulating insulin transport across the BBB (20, 37). Research has identified ALP as a receptor for engineered viral vectors, facilitating their transport across the BBB (38). Elevated ALP levels may lead to abnormalities in the BBB regarding protein transportation. Endothelial ALP is expressed as the BBB matures and is absent from non-BBB vessels, indicating it is a key component of the “enzymatic barrier” in cerebral microvessels (37). ALP enzymatic activity regulates the transport of small molecules, its inhibition reduces substrate uptake by keeping transporters in a phosphorylated, inactive state (39). In the context of post-EVT ischemia–reperfusion injury, excessive ALP activity may exacerbate endothelial dysfunction, further increasing BBB permeability and elevating the risk of hemorrhagic transformation—a key determinant of unfavorable prognosis. In addition, both stroke itself and endovascular thrombectomy procedures may augment neuroinflammatory responses; pro-inflammatory cytokines suck as TNF-α (tumor necrosis factor-α) and IL-6 (interleukin-6) can in turn further disrupt BBB integrity (40).

Our study specifically focuses on patients with anterior circulation large vessel occlusion, as posterior circulation strokes were excluded due to their significantly higher mortality rates and the relatively limited sample size of such cases in our cohort. While the systemic nature of ALP-related pathophysiological mechanisms—such as vascular calcification and inflammation—suggests that the observed association could plausibly extend to posterior circulation stroke patients, this remains speculative and requires validation in dedicated studies. Furthermore, although our cohort reflects a real-world anterior circulation endovascular thrombectomy population, the single-center and retrospective nature of our study may still limit extrapolation to broader populations, including non-Asian cohorts and those with underrepresented comorbidities.

This study has several limitations. Despite adjusting for many covariates, potential confounders such as smoking and drinking history, pre-stroke medication (e.g., statins, antiplatelet drugs), and in-hospital complications (e.g., pneumonia) were not available for adjustment, which might have introduced residual confounding. Additionally, although we adjusted for liver enzymes and inflammatory markers in our analyses, we did not explicitly exclude patients based on clinical diagnoses of severe liver disease, active malignancy, or severe systemic infections. These conditions represent primary sources of serum ALP elevation, and their inclusion in the study may introduce confounding and heterogeneity, thereby compromising the interpretation of the association between ALP and prognosis following AIS thrombectomy. Non-stroke-related ALP elevation can distort the true relationship between ALP and clinical outcomes relative to “stroke-specific ALP” (linked to vascular calcification, neuroinflammation, or BBB dysfunction), while comorbidity-associated factors (e.g., advanced age, coagulopathy) independently influence post-thrombectomy outcomes and increase interindividual ALP variability (41, 42). Furthermore, the distinct biological effects of bone-specific and liver-specific ALP isoenzymes are obscured by this heterogeneous spectrum of ALP elevation (6), which weakens the robustness of our observed association and limits the generalizability of our findings to AIS patients without such comorbidities. Lastly, the single ALP measurement obtained at admission merely captures a static snapshot of the enzyme level prior to intervention, failing to account for potential fluctuations induced by subsequent key pathophysiological processes. These include procedural-related traumatic stress during endovascular thrombectomy, as well as post-operative complications such as infections—factors that may exert substantial impacts on systemic ALP activity (43, 44). Therefore, future prospective studies should incorporate serial ALP measurements (e.g., at admission, 24–48 h post-procedure, and during follow-up) to capture its dynamic trajectory throughout the acute phase of stroke management.

## 5 Conclusion

In conclusion, our study suggests that higher ALP levels at admission are independently associated with unfavorable prognosis in AIS patients treated with ET. Further large-scale, multicenter prospective studies are warranted to confirm these findings.

## Data Availability

The raw data supporting the conclusions of this article will be made available by the authors, without undue reservation.
