# Supplementary material for: Serum alkaline phosphatase levels at admission are associated with unfavorable prognosis in acute ischemic stroke patients undergoing endovascular thrombectomy
Source: Front Neurol. 2026 Feb 17;17:1738653. doi: 10.3389/fneur.2026.1738653 (PMC12953080; doi:10.3389/fneur.2026.1738653)
Supplement: Supplementary file 1 [file Table_1.DOCX]

Table S1 Baseline comparison of included and excluded patients.

|  | Included (n = 385) | Excluded (n = 151) | *P*-value |
| --- | --- | --- | --- |
| Sex, n (%) |  |  | 0.573 |
| Male | 245 (63.6) | 100 (66.2) |  |
| Female | 140 (36.4) | 51 (33.8) |  |
| Age, years, Mean ± SD | 66.6 ± 12.5 | 65.5 ± 12.8 | 0.363 |
| EH, n (%) | 196 (50.9) | 66 (44.3) | 0.170 |
| DM, n (%) | 78 (20.3) | 24 (16.1) | 0.274 |
| Dyslipidemia, n (%) | 24 (6.2) | 5 (3.4) | 0.188 |
| AF, n (%) | 178 (46.2) | 66 (44.3) | 0.687 |
| CAD, n (%) | 53 (13.8) | 29 (19.6) | 0.095 |
| Cause, n (%) |  |  | 0.227 |
| Atherosclerotic | 159 (41.3) | 63 (41.7) |  |
| Cardioembolic | 173 (44.9) | 59 (39.1) |  |
| Others | 53 (13.8) | 29 (19.2) |  |
| Baseline NIHSS, Mean ± SD | 17.9 ± 8.0 | 17.4 ± 7.2 | 0.565 |
| Baseline ASPECTS, Mean ± SD | 7.7 ± 1.5 | 7.5 ± 2.0 | 0.194 |
| IV thrombolysis, n (%) | 133 (34.5) | 54 (36.5) | 0.674 |
| Occlusion site, n (%) |  |  | 0.866 |
| ICA | 129 (33.5) | 53 (35.1) |  |
| M1 | 240 (62.3) | 93 (61.6) |  |
| M2 | 16 (4.2) | 5 (3.3) |  |
| OTP time, minutes, Mean ± SD | 278.6 ± 125.3 | 292.2 ± 131.1 | 0.266 |

Note: EH, hypertension; DM, diabetes mellitus; AF, atrial fibrillation; CAD, coronary artery disease; NIHSS, National Institutes of Health Stroke Scale; ASPECTS, Alberta Stroke Program Early CT Score; IV, intravenous; ICA, internal carotid artery; M, middle cerebral artery; OPT, onset to groin puncture time.
