# Supplementary figures and images for: Serum alkaline phosphatase levels at admission are associated with unfavorable prognosis in acute ischemic stroke patients undergoing endovascular thrombectomy
Source: Front Neurol. 2026 Feb 17;17:1738653. doi: 10.3389/fneur.2026.1738653 (PMC12953080; doi:10.3389/fneur.2026.1738653)

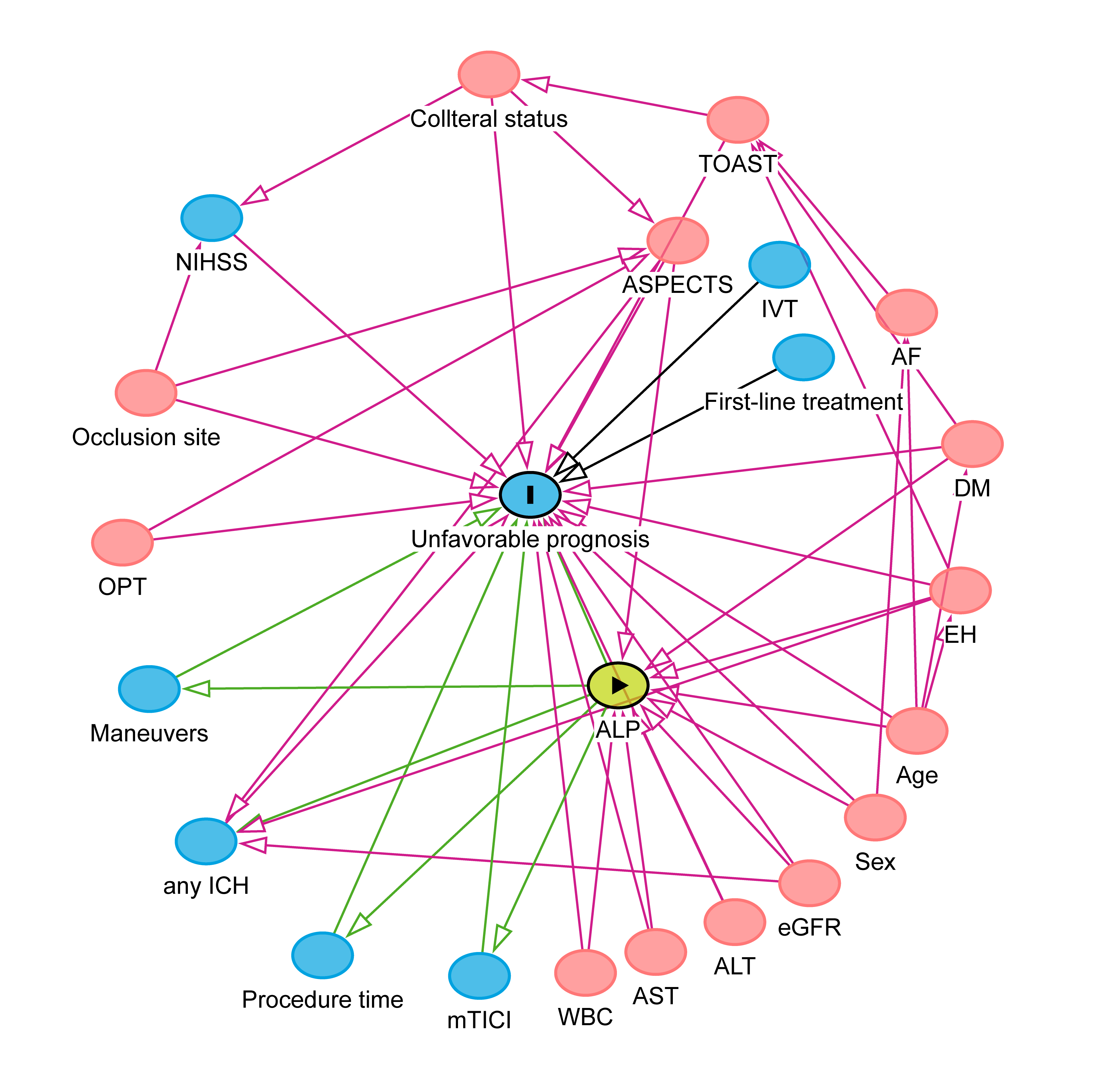

Supplement: Supplementary file 2 [file Image_1.TIF]
